# Proposal of layered mental healthcare for mental well‐being

**DOI:** 10.1049/htl2.12012

**Published:** 2021-05-13

**Authors:** Masashi Kiguchi, Stephanie Sutoko, Hirokazu Atsumori, Ayako Nishimura, Akiko Obata, Tsukasa Funane, Hiromitsu Nakagawa, Masashi Egi, Hiroyuki Kuriyama

**Affiliations:** ^1^ Center for Exploratory Research Hitachi, Ltd. Kokubunji Tokyo Japan; ^2^ Lumada Data Science Lab. Hitachi, Ltd. Kokubunji Tokyo Japan; ^3^ Central for Technology Innovation Hitachi, Ltd. Kokubunji Tokyo Japan; ^4^ Global Center for Social Innovation Hitachi, Ltd. Kokubunji Tokyo Japan

## Abstract

A new concept, ‘Layered mental healthcare’ for keeping employees mental well‐being in the workplace to avoid losses caused by both absenteeism and presenteeism is proposed. A key factor forming the basis of the concept is the biometric measurements over three layers, i.e., behaviour, physiology, and brain layers, for monitoring mental/distress conditions of employees. Here, the necessity of measurements in three layers was validated by the data‐driven approach using the preliminary dataset measured in the office environment. Biometric measurements were supported by an activity tracker, a PC logger, and the optical topography; mental/distress conditions were quantified by the brief job stress questionnaire. The biometric features obtained 1 week before the measurement of mental/distress scores were selected for the best regression model. The feature importance of each layer was obtained in the learning process of the best model using the light graded boosting machine and was compared between layers. The ratio of feature importance of behaviour:physiology:brain layers was found to be 4:3:3. The study results suggest the contribution and necessity of the three‐layer features in predicting mental/distress scores.

## 1 INTRODUCTION

Organisation for Economic Co‐operation and Development (OECD) reported that mild‐to‐moderate mental disorders (e.g. anxiety, depression) affect around 20% of the working‐age population [1]. Mental disorders bring significant concerns for economic development and social welfare [2]. For example, U.S. workers suffering from depression cost employers an additional 31 billion dollars each year due to lost productive time [3]. The Japanese Government estimated that the economic and social loss from suicides and mental disorders were at least 2.7 trillion yen (about 25 billion dollars), which is equivalent to 0.7 per cent of the GDP in 2009 [4]. Furthermore, depression was highly associated with the presenteeism [5] causing the health‐related productivity loss during paid hours [6, 7, 8]. Companies need systems for maintaining their employees mental well‐being to avoid both absenteeism and presenteeism. A low‐cost and easy solution is demanded in workplaces. We here propose a new concept, ‘layered mental healthcare’ based on biometric measurements.

## 2 LAYERED MENTAL HEALTHCARE

**FIGURE 1:**
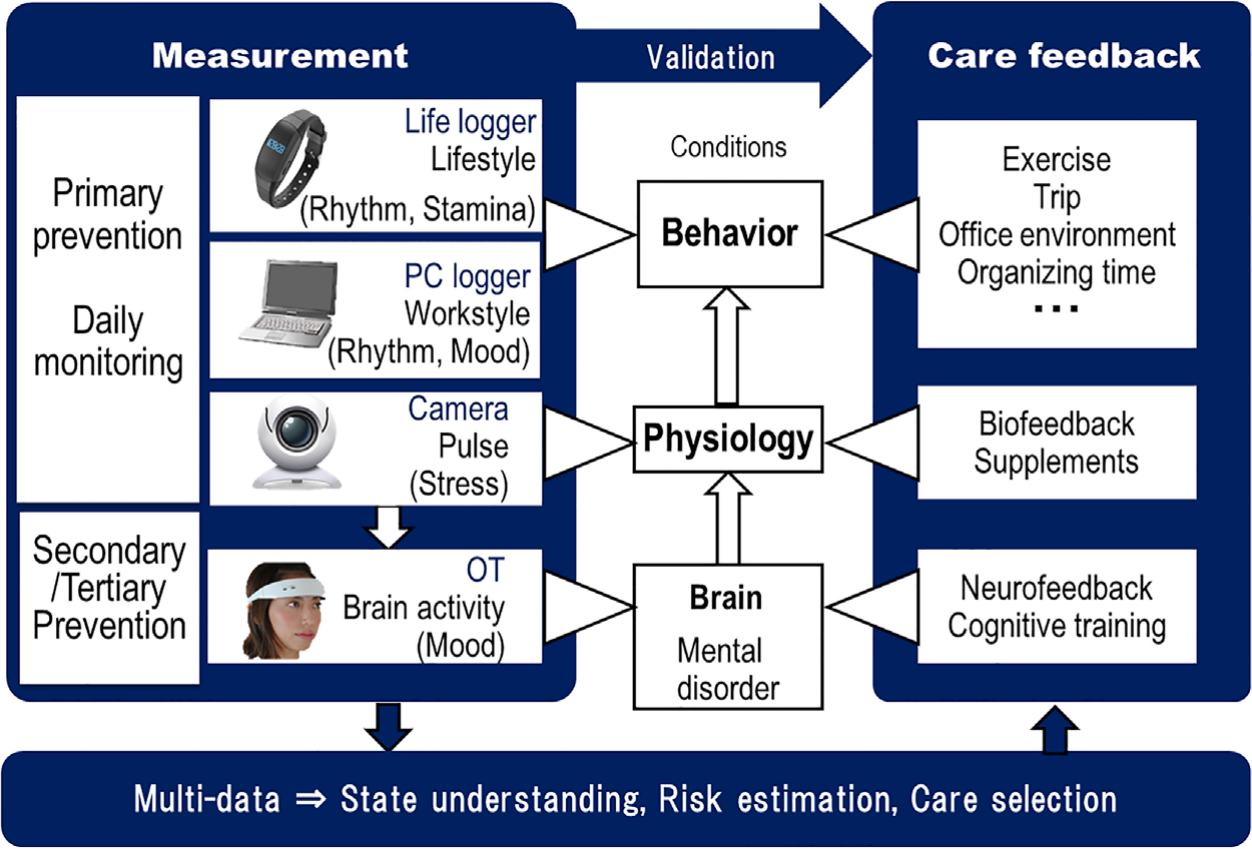
Concept of ‘Layered mental healthcare’. OT is the optical topography, i.e., functional near‐infrared spectroscopy device

‘Layered mental healthcare’, is a concept for screening and managing the mental well‐being in workplaces besides the medical diagnosis, treatments, and cares (Figure 1). The mental disorder originates from the brain, affects physiology, such as heart rate and blood pressure, and alternates behaviour. The changes in the three layers of behaviour, physiology, and brain should be measured to monitor the mental/distress conditions and estimate the risk. In this concept, measurement devices were designed to be wearable; non‐clinical cares for each layer can also be expected. For example, leisure activities (e.g. travel, exercise, entertainment), supplement administrations, and neurofeedback were suggested for the disrupted behaviour, physiology, and brain layers, respectively. Condition monitoring is also helpful for evaluating the effectiveness of cares with low costs. Furthermore, the management of working hours, conditions, and environment can be recommended to employers, depending on the employees conditions.

A lot of biometric measurements have been used for monitoring distress and also both bio‐and neuro‐feedbacks for stress coping [9]. The concept of layer is introduced here to cover a variety of preceding symptoms. The measurements/cares are selected depending on the required accuracy/efficacy and acceptable cost. Physiological markers and behaviours were collected using wearable sensors and mobile phones for the classification of high or low stress [10].

A multimodal system using the electroencephalography (EEG), hemoencephalography (HEG), and heart rate variability (HRV) was reported [11]. A multimodal system over the three layers was a new point of this letter. However, it is not clear if the three‐layer measurements are significantly needed for monitoring mental/stress conditions. The purpose of this letter is to investigate the contribution of the three‐layer measurements in predicting mental/distress conditions. The concept of ‘Layered mental healthcare’ was validated using the data‐driven technique.

## 3 DEVICES, TRIAL IN OFFICE AND ANALYSIS

The devices were selected for the validation of the concept according to the previous studies as follows. For the behaviour layer, a home‐made wrist‐band type activity tracker (Life logger) and the PC logger recording event‐logs of keyboard and mouse operations were used for monitoring the lifestyle [12] and the workstyle [13], respectively. The wrist‐band type activity tracker had a triaxial accelerometer to provide the number of steps, the index of exercise intensity in metabolic equivalents, etc. By using the key/mouse data recorded over the daily course, a fractal dimension was obtained as a slope of fitted line for cumulative distribution vs time‐interval of key/mouse events graph [13]. The fractal dimension represents a kind of rhythm of PC operations, which depends on the mood state. For the physiology layer, the HRV, a well‐known biomarker for stress, was measured [10, 11]. Even though a camera can accommodate the HRV measurement (Figure 1), the wearable optical topography (OT, model HOT‐1000, NeU) was currently used to measure the HRV derived from forehead pulses as well as the brain measurement. The ‘LF/HF’ ratio reflecting the sympatho‐vagal balance was calculated. For the brain layer, the hemodynamic change in the dorsolateral prefrontal cortex during the spatial and verbal delayed matching tasks (working memory function) was observed using OT [14, 15]. The difference of oxygenated hemoglobin changes between the verbal and spatial delayed matching tasks which are well correlated with scores of the profile of mood state.

**FIGURE 2:**
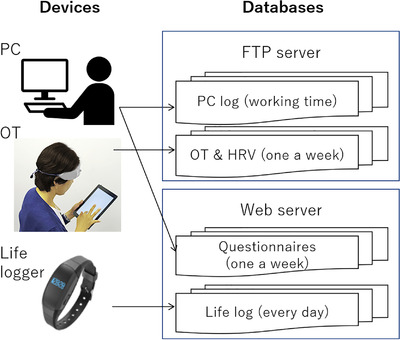
Configuration diagram of data accumulation system for the trial in office. Recorded data of PC log, OT including HRV, answers of questionnaires, and life log were accumurated in the databases on the servers. Each record frequency is shown in parenthesis

Figure 2 shows the data accumulation system used in the trial for validating the ‘Layered mental healthcare concept’. All data measured using the devices were accumulated in the database servers. Thirty‐nine healthy volunteers (32 males, 7 females, 43.7 ± 8.9 years old) with no history of mental disorders participated in this study; the measurement was lasted for about four months [16]. The data from the volunteers were obtained according to the regulations set forth by the internal review board at the Central Research Laboratory, Hitachi, Ltd., following the receipt of their written informed consent. The PC logger was installed on each PC used by each participant in the office. The log data were automatically recorded during the working time. The fractal indexes of key and mouse operations in a day were calculated from the logs. The life logger was worn all day including holidays. Steps, exercise, intensity in metabolic equivalents, and sleeping time in a day were obtained. Once a week at the relatively same time, the OT and questionnaire (Kessler 6 (K6), twenty‐nine questions of Brief Job Stress (BJS) Questionnaire [17, 18, 19]) measurements were performed. The participants wore the OT headset and performed the delayed matching tasks which appeared in the tablet PC. The cerebral blood changes associated with brain activity during the task and HRV data were recorded in the tablet PC. Three cardiac features and eleven features of brain activity during tasks were obtained by OT measurement. Each participant answered the questionnaires shown in a web browser. BJS provides scores of anxiety, depression, fatigue, irritation, physical stress, vigor, and the total of them. The worse condition was marked by lower scores. K6 was not used in this analysis.

Data preprocessing were performed for all of the accumulated data before the machine learning process. Each features obtained from the PC logger and the life logger was averaged for a week. The outliers of feature data (< –3 σ or > 3 σ) were removed. Independent features with cross‐correlation less than 0.6 were selected to avoid the multicollinearity. The weekly data with missing records due to forgetting, absence, and business trip were removed. No standardisation of data was performed. Finally, 96 weekly records of feature data were obtained for each target variable (i.e. BJS scores). Furthermore, weekly feature records were obtained some weeks before and after the week; the targets measured were prepared by temporally shifting the feature data.

Light Gradient Boosting Machine (LGBM) [20, 21] was used for obtaining the feature importance based on gain for each target variable. The nested cross‐validation (cv) was performed to obtain the best models using 10‐fold outer cv and to tune hyper‐parameters of LGBM with Optuna [22] using 5‐fold inner cv. The objective function was L2 loss (the least square errors) in the case of regression. The best models were selected according to the adjusted R^2^ averaged across targets, because the valid sample size depended on the set of records. Then, each feature importance for each target was obtained using the set of records that provided the best models.

## 4 RESULTS AND DISCUSSION

The selected features together with the results of Shapiro Wilk normality test are shown in Table 1. The simplest feature, ‘Ped’ and ‘Keylog’ were selected among behaviour features. The physiological features of ‘HRstdev’ and “LF/HF” were favoured rather than the heart rate itself. For the brain layer, ‘OT_sv_L’ and ‘OT_sv_rt’ which are correlated with the mood state and the ability of brain function were selected. The comparison of the selected features between layers was performed. The target variables were not normally distributed because those variables are discrete scores. Some features are also not normally distributed; the small sample size might cause these non‐normal distributions. Therefore, the regression trees, which do not require the assumption of normal distribution for variables were used for making models.

**TABLE 1:** Features selected as explanatory variables. ‘Non’ in Normality means that the normal distribution of feature variable was rejected with a significance level of 5% by Shapiro Wilk test

Feature	Device	Description	Normality (p‐value)
Ped	Life logger	Steps in a day	– (0.094)
Keylog	PC logger	Fractal index of key operation in a day	– (0.37)
HRstdev	OT	Standard deviation of heart rate during the task	Non (8.4E‐6)
LF/HF	OT	Power ratio of low (0.04–0.15 Hz) to high (0.15‐0.4 Hz) frequency of pulse cycle	Non (1.5E‐7)
OT_sv_L	OT	Difference of left prefrontal cortex activity between during verbal and spatial working memory tasks	Non (0.0046)
OT_sv_rt	OT	Difference of response time between to verbal and spatial working memory tasks	Non (0.0030)

**FIGURE 3:**
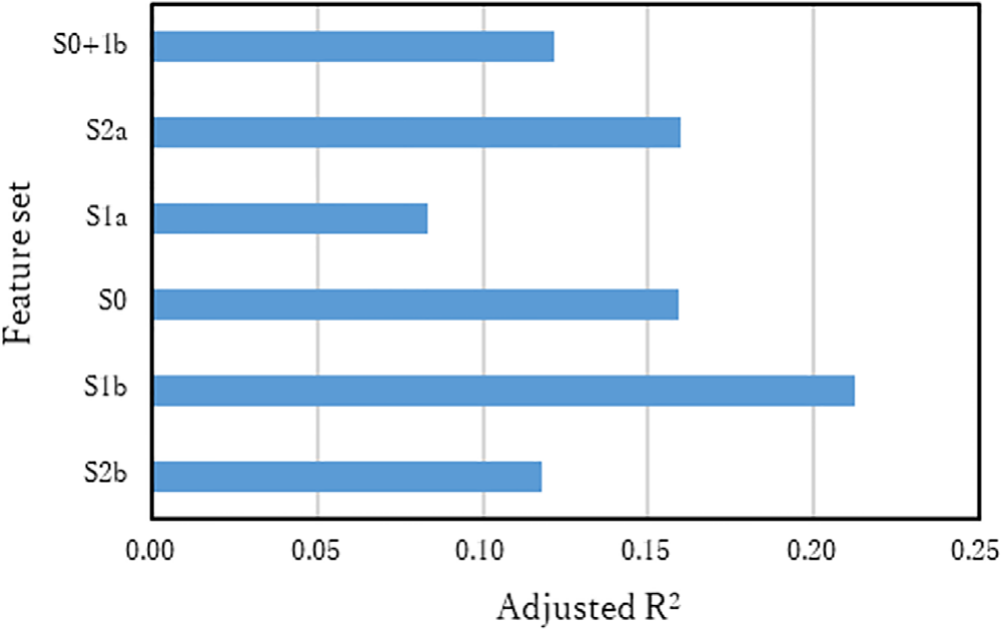
Adjusted R^2^ averaged across mental scores of BJS for each feature set

Because the features objectively measured using the devices may change earlier/later than the mental/distress scores subjectively complained, the combination of features and target values obtained in the different week were also tested. Figure 3 shows the comparison of the best adjusted R^2^ averaged across target variables between the set of records shifted weeks. The sets of S1b, S2b, S1a, and S2a were combinations of the target variable and the feature variables obtained 1 and 2 weeks prior to and 1 and 2 weeks after the measurement of target variables, respectively. The feature variables in set S0 were obtained in the week of target variable measurement. Meanwhile, S0+1b had both feature variables of S0 and S1b. The adjusted R^2^s of S1b and S0 were placed in the first and second rank, respectively. These results suggested that the mental modulation on three‐layer features was objectively observed prior to the target variables. These results are reasonable because the questionnaires asked mental conditions within the last 1 week. Therefore, through the three‐layer measurement, the mental condition can be indicated early, and this early detection becomes one of the advantages of the current concept.

**FIGURE 4:**
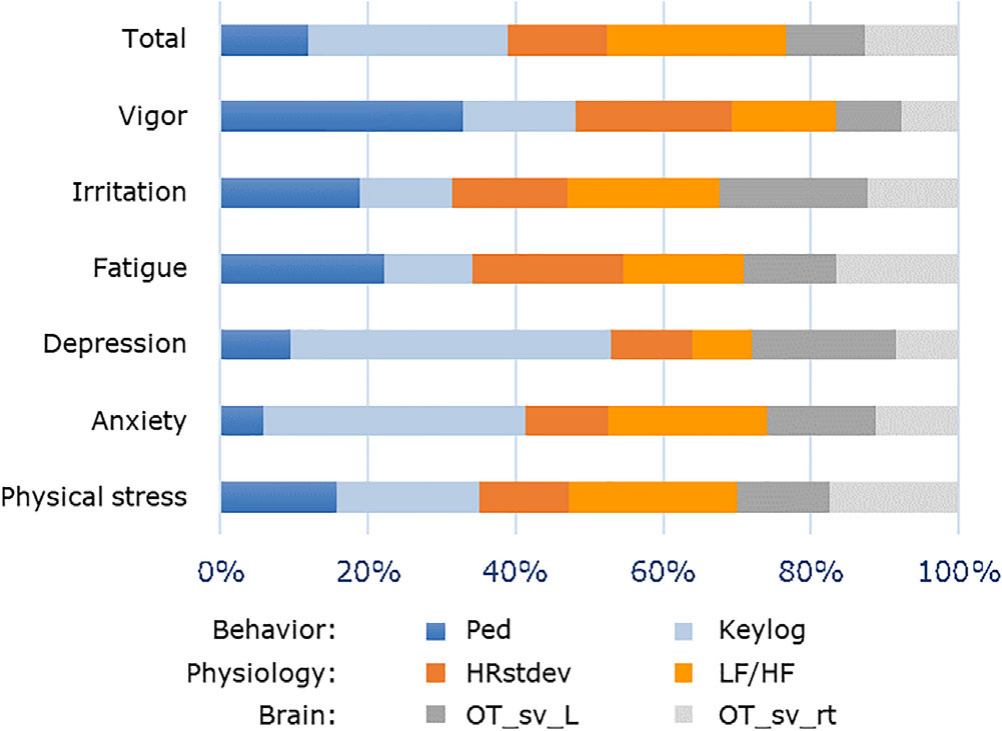
Percentage of feature importance in the behaviour, physiology, and brain layers for predicting the mental scores of BJS questionnaire using Light Graded Boosting Machine with explanatory variables obtained 1 week prior to the measurement of mental scores

Figure 4 shows the ratios of feature importance (‘gain’) for each target variables using the best models with S1b. The feature importance is a measure of the degree of improvement in the predictive accuracy of the model by branching at the feature quantity. It is used to assess the relative contribution of the feature quantity to the model. For example, the 1‐week shifted keylog feature highly contributed to the depression. Each layer importance was calculated by the summation of feature ratios in each layer for each target variable. The ratio of layer importance averaged across the seven target variables were calculated as,

Behaviour:Physiology:Brain = 4:3:3.

The feature importances are comparable across layers. Therefore, the three‐layer measurements were important for estimating the mental/distress conditions.

**FIGURE 5:**
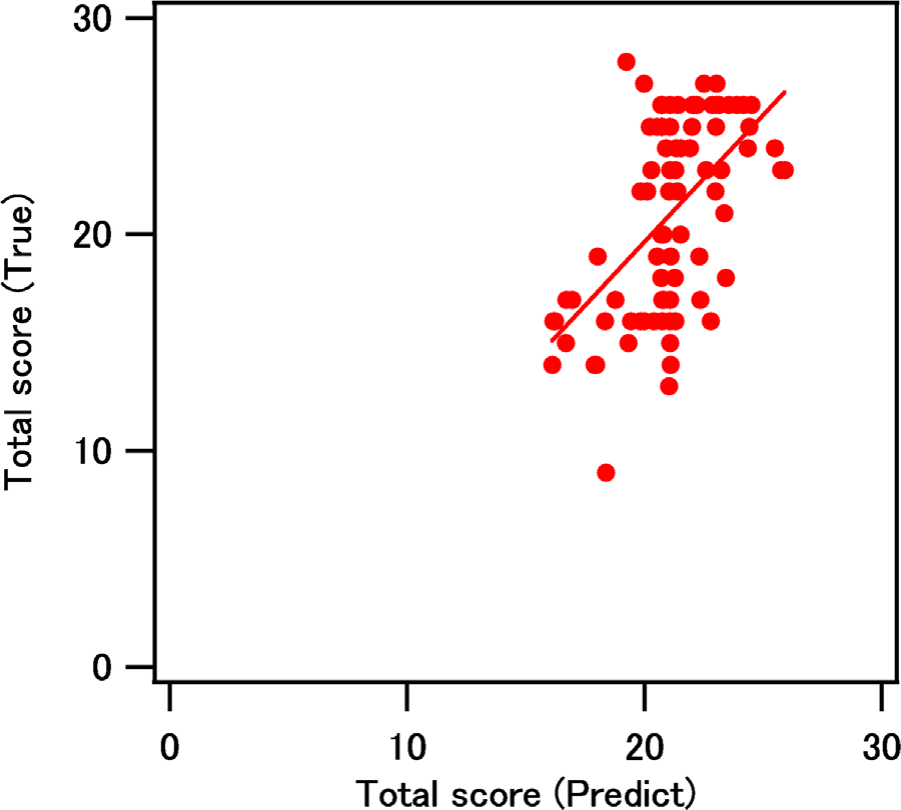
Relationship between the predicted and true values of the total BJS questionnaire scores. Fitted line is obtained by the linear regression using both predicted and true values

The relationship between the total BJS scores predicted by the best models and the corresponding test data is shown in Figure 5 as a reference example. The Pearson correlation coefficients for the mental/distress scores of BJS were under the approximate condition of normal distribution in Table 2. These values are relatively acceptable except for the irritation, considering the small variance data for healthy participants. The development of the best models for prediction is another topic. Because the regression tree was used for quantitatively investigating the contribution of features in three layers, it did not necessarily provide the best model for prediction. The prediction accuracy is potentially improved by optimising the explanatory variables, introducing the interaction effects, and using suitable regressors, such as the support vector machine and the neural network. The features in the physiology layer obtained during the task once a week using OT were different from the features obtained using the heart rate monitor during the resting state. A daily and continuous measurement using the mobile pulse meter may provide better prediction results. The models depending on the individual/state will be led through more trials. In order to show the efficiency of the ‘Layered mental healthcare’ concept, a protocol of measurements should consider the trade‐off between accuracy and cost; the care recommendation methods are expectedly developed in the next step.

**TABLE 2:** Pearson correlation coefficients between the predicted and true values for each mental score of BJS

Mental/distress score of BJS	Pearson correlation coefficient
Total	0.55
Irritation	0.32
Fatigue	0.48
Depression	0.57
Anxiety	0.55
Physical stress	0.55

## 5 CONCLUSION

By using the data obtained during the 14‐week trial in office, the best models for predicting mental scores of BJS were obtained using LGBM. The biometric features obtained 1 week prior to the measurement of the mental scores provided the best models. The ratio of feature importance for the layers of behaviour:physiology:brain was 4:3:3. These results suggested that the prediction contribution of each layer was comparable. Therefore, the three‐layer measurement was necessary for monitoring the mental/distress conditions. Because the mental/distress conditions are risk factors of mood disorders, the three‐layer measurement is potentially helpful for an early detection before the onset of clinical symptoms of mood disorders. The measurement purposes can be extended for managing mental conditions and preventing mood disorders by recommending suitable cares. The current results also confirmed and validated the benefits of the ‘Layered mental healthcare’ concept in realising the mental well‐being in workplaces.
